# Evolution of the *SPATULA/ALCATRAZ* gene lineage and expression analyses in the basal eudicot, *Bocconia frutescens* L. (Papaveraceae)

**DOI:** 10.1186/s13227-017-0068-8

**Published:** 2017-03-15

**Authors:** Cecilia Zumajo-Cardona, Barbara Ann Ambrose, Natalia Pabón-Mora

**Affiliations:** 1 Instituto de Biología, Universidad de Antioquia, Medellín, 1226, Colombia; 2 New York Botanical Garden, Bronx, NY 10458 USA; 3 City University of New York, New York, NY 10016 USA

**Keywords:** *ALCATRAZ*, Basal eudicots, *Bocconia frutescens*, Fruit development, *paleoSPT/ALC*, Papaveraceae, *SPATULA*

## Abstract

**Background:**

*SPATULA* (*SPT*) and *ALCATRAZ* (*ALC*) are recent paralogs that belong to the large bHLH transcription factor family. Orthologs of these genes have been found in all core eudicots, whereas pre-duplication genes, named *paleoSPATULA/ALCATRAZ*, have been found in basal eudicots, monocots, basal angiosperms and gymnosperms. Nevertheless, functional studies have only been performed in *Arabidopsis thaliana*, where *SPT* and *ALC* are partially redundant in carpel and valve margin development and *ALC* has a unique role in the dehiscence zone. Further analyses of pre-duplication genes are necessary to assess the functional evolution of this gene lineage.

**Results:**

We isolated additional *paleoSPT/ALC* genes from *Aristolochia fimbriata*, *Bocconia frutescens*, *Cattleya trianae* and *Hypoxis decumbens* from our transcriptome libraries and performed phylogenetic analyses. We identified the previously described bHLH domain in all analyzed sequences and also new conserved motifs using the MEME suite. Finally, we analyzed the expression of three *paleoSPT/ALC* genes (*BofrSPT1/2/3*) from *Bocconia frutescens*, a basal eudicot in the Papaveraceae. To determine the developmental stages at which these genes were expressed, pre- and post-anthesis carpels and fruits of *B. frutescens* were collected, sectioned, stained, and examined using light microscopy. Using in situ hybridization we detected that *BofrSPT1/2/3* genes are expressed in floral buds, early sepal initiation, stamens and carpel primordia and later during fruit development in the dehiscence zone of the opercular fruit.

**Conclusions:**

Our expression results, in comparison with those available for core eudicots, suggest conserved roles of members of the *SPT/ALC* gene lineage across eudicots in the specification of carpel margins and the dehiscence zone of the mature fruits. Although there is some redundancy between *ALC* and *SPT*, these gene clades seem to have undergone some degree of sub-functionalization in the core eudicots, likely by changes in cis regulatory regions and to some extent in coding sequences, at least in Brassicaceae. Our results also indicate that in *Bocconia frutescens*, paleo*SPT/ALC* genes may play a role in early floral organ specification that was subsequently lost in core eudicot lineages.

**Electronic supplementary material:**

The online version of this article (doi:10.1186/s13227-017-0068-8) contains supplementary material, which is available to authorized users.

## Background

Plants provide a great experimental model to assess functional evolution after gene duplication as most plant genomes have been shaped by ancient whole genome duplication (WGD) events [1, 2]. Gene and genome duplications can result in mutational loss of duplicates, redundancy, the acquisition of new roles due to changes in regulatory or protein interactions (neofunctionalization) or the redistribution of functions among paralogs by uncoupling of regulatory elements (sub-functionalization) [2–5]. Functional diversification after gene and genome duplication has received considerable attention in polyploid crops, but can also be assessed by comparing gene functions at different time points in the phylogeny prior to and after major WGD in angiosperms [2, 6]. Paleopolyploidy has been traced back to WGD occurring before the diversification of angiosperms, prior to the origin of core eudicots, within the Brassicales and Solanales, and concomitant with monocot diversification [7–9]. In this framework, basal eudicots have become a unique reference for assessing gene functional evolution in core eudicots, the latter include 75% of flowering plant species with unique paleopolyploidy events, whereas the former include species with pre-duplication genes, often single copy, most of the time exemplifying the ancestral role prior to the WGD events.

The basic/helix-loop-helix (bHLH) proteins are a superfamily of transcription factors that has been better characterized in animals than in plants [10, 11]. However, the *Arabidopsis* genome possesses at least 147 bHLH protein-encoding genes, making this, one of the largest transcription factor families in this model species, likely having important unexplored functions [12]. The key developmental transcription factors *ALCATRAZ* (*ALC*) and *SPATULA* (*SPT*) are two closely related factors that belong to this transcription factor family [12–15]. *SPATULA* (*SPT*) is expressed in developing carpel margins, leaves and petals, as well as the dehiscence zone of fruits and anthers [16]. In *Arabidopsis thaliana, SPT* is important for carpel margin development which impacts proper carpel fusion, transmitting tract development, as well as style and stigma development [13, 16–19]. Loss of function phenotypes in *spt* mutants exhibit defective carpel margin fusion, particularly at the distal-most portion of the congenitally fused, bicarpellate gynoecium [17, 19]. *ALCATRAZ* is turned on in the petal margins, in the stamens, stigmas and carpellary margins and later in development, at the layer of non-lignified cells in the silique or silicle [20, 21]. *ALC* is required for proper fruit dehiscence zone development, in particular as it specifies the identity of the separation layer, rich in cell-wall degrading enzymes [20, 22]. In addition, *SPT* and *ALC* proteins are able to form heterodimers and have redundant roles in gynoecium development. More specifically, they are key factors during carpel and valve (fruit wall) margin development, as shown by the *spt/alc* double mutant, which exhibits increased severity in the carpel separation as well as defects in style and stigma patterning and the histogenesis of the valve margin, and the dehiscence zone [21]. However, while *SPT* overexpression can fully compensate *alc* defects during fruit development and dehiscence, *ALC* overexpression can only partially compensate for *spt* defects by increasing the short fruit size and restoring, to some extent, apical fusion between the carpels [21]. SPATULA also interacts with HECATE1 (HEC1), HEC2, and HEC3, all bHLH transcription factors involved in septum, transmitting tract and stigma development [23, 24]. On the other hand, ALCATRAZ interacts with ALC interacting protein1 (ALC1), a protein expressed in vascular and mesocarp cells in *Arabidopsis* [25]. Thus, it is likely that despite the fact that they act partly redundantly in early gynoecium patterning and late fruit development, there is some specialization due to changes in expression patterns and protein interactions.

The current working model for a gene regulatory network in fruit development includes both *SPT* and *ALC* functioning downstream of two MADS-box genes, *FRUITFULL* and *SHATTERPROOF* which establish valve and valve margin identity, thus delineating the dehiscence zone [20, 26, 27]. *FRUITFULL* (*FUL*) controls valve identity, as shown by the prematurely exploded fruits with bursting seeds of the *ful* mutants [26]. *SHATTERPROOF1/2* (*SHP1/2*) controls the dehiscence zone differentiation by promoting adjacent lignified and unlignified cells in the valve margin [27]. In the *ful* mutant, *SHP* expands its expression to the fruit valves while, in the *shp1/2* mutant, *FUL* does not expand its expression to the valve margin [27]. Consistent with the proposed interactions, in the *alc* mutant, the expression of both upstream transcription factors *SHP1/2* and *FUL* is not altered [27]. Both, *SPT* and *ALC,* are activated by *SHP1/2* [20], that in turn are negatively regulated by *FUL* [28]. However, this genetic network is restricted to *Arabidopsis,* as a number of these genes is not conserved in all angiosperms due to lineage specific duplications mostly occurring in Brassicaceae and core eudicots [29].

Phylogenetic analyses across seed plants have shown that *SPATULA* and *ALCATRAZ* belong to paralogous clades resulting from a duplication event that occurred prior to the diversification of the core eudicots; in non-core eudicots, pre-duplication genes are referred to as the *paleoSPT/ALC* genes [29]. Comparisons between SPT and ALC sequences across flowering plants reveal that the bHLH domain is highly conserved. Nevertheless, SPT proteins have a conserved acidic domain and amphipathic helix N terminal to the bHLH domain. The amphipathic helix but not the acidic domain has been identified in ALC proteins [13, 21, 29, 30]. *paleoSPT/ALC* orthologs have the acidic domain and exhibit conserved key functional residues in the bHLH domain, suggesting that paleoSPT/ALC may have similar downstream targets as the Arabidopsis SPT and ALC [29].

Functional and expression analyses of *SPT* and *ALC* have only been performed in a few core eudicots including *Arabidopsis thaliana*, *Lepidium campestre*, *Fragaria vesca* and *Prunus persica* [13, 20, 30–32]. In *Lepidium campestre* (Brassicaceae), *ALC* is expressed in the dehiscence zone of the fruit, suggesting that the function of *ALC* is conserved in several members of Brassicaceae exhibiting the distinct silique or silicle fruit types [32]. In *Fragaria vesca,* functional studies have shown that *FaSPT*, the *SPT* ortholog, is involved in strawberry development, as *faspt* fruits in early stages exhibited reduced size [31] similar to a role for Arabidopsis *SPT* in regulating organ size [33]. In *Prunus persica* (peach), expression analysis of the *SPT* ortholog, *PPERALCATRAZ/SPATULA* (*PPERALC/SPT*), showed that it is expressed in the perianth, ovary and later in the endocarp margins as well as in leaves [30].

**Fig. 1:**
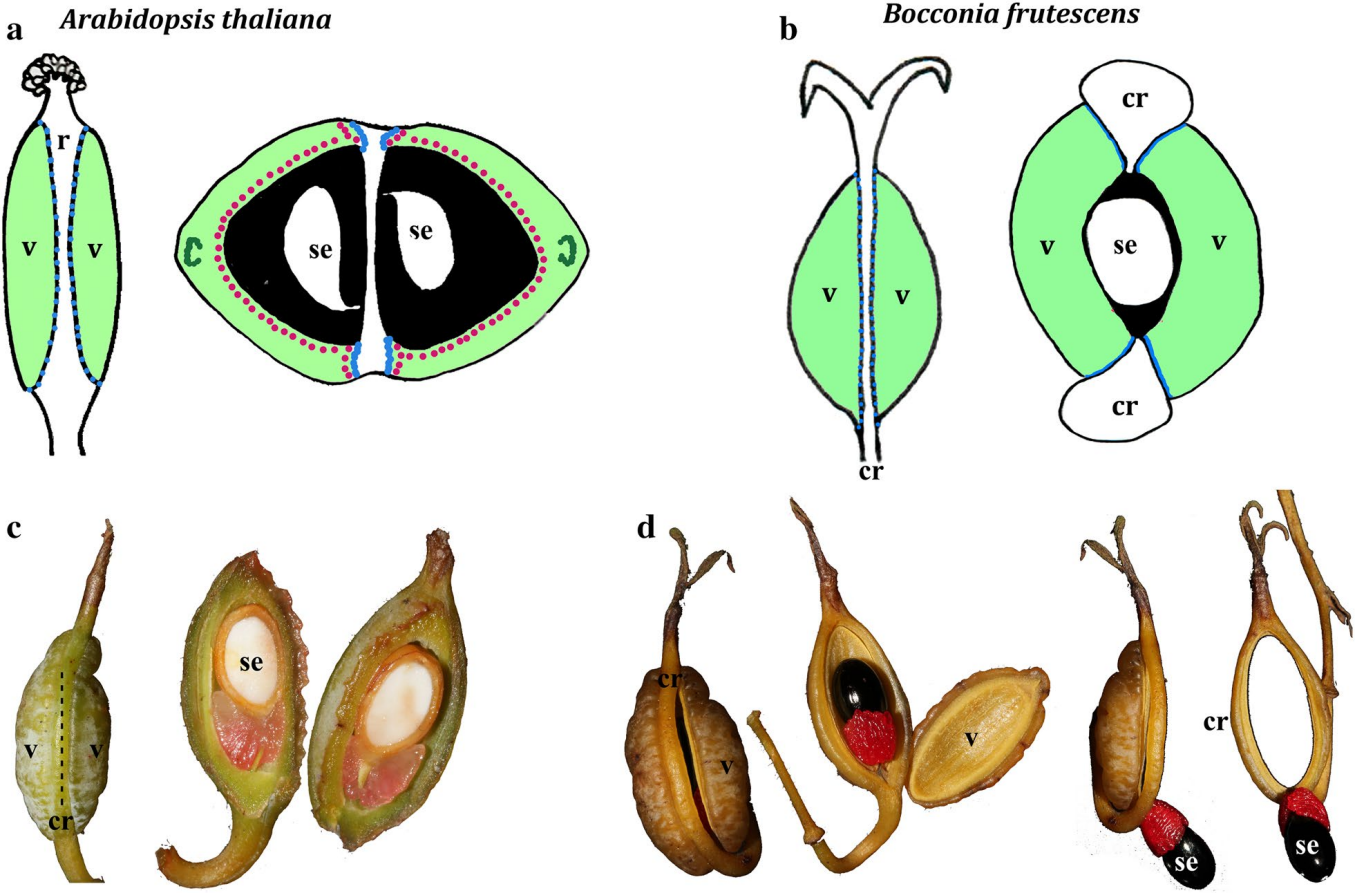
Schematic representation in transverse section of **a** *Arabidopsis thaliana* and **b** *Bocconia frutescens* fruits. *Black*, locules; *dark green*, main carpel vascular bundles; *light green*, carpel wall; *pink*, lignified tissue; *blue*, dehiscence zones; *cr* commissural ring, *se* seed, *v* valve; *arrows* point to the dehiscence zones in sections. **c** Immature fruit (6–7 mm diameter) of *B. frutescens* closed to the *left* and cut opened to the *right*. **d** Mature fruit (1 cm diameter) of *B. frutescens,* closed to the left and in successive opening stages to the right. Note the ring-like structure remaining after valve separation. This figure has been modified from Pabón-Mora et al. [29]

As expression and functional analyses in this gene lineage are restricted to only a few core eudicot species, it is difficult to predict the functional evolution of this gene lineage. Thus, to better understand the evolution of this gene lineage we here report: (1) expanded sampling of *SPT/ALC* homologs across flowering plants; (2) the analysis of coding sequences of SPT and ALC genes prior to and after the core eudicot duplication to identify conserved regions between the pre- and post-duplication homologs that may help predict putative shifts in protein function; (3) the comparisons of expression patterns of pre- and post-duplication homologs using the online available tools for model species (i.e., eFP browser in *Arabidopsis thaliana*, *Medicago truncatula*, *Solanum lycopersicum*, *S. tuberosum* and *Oryza sativa*; and (4) the expression analyses of selected *paleoSPT/ALC* genes in *Bocconia frutescens* (Papaveraceae, Basal eudicot). We chose *B. frutescens* because: 1) it is a member of the basal eudicots; thus, it possesses paleo*SPT/ALC* genes predating the core eudicot duplication; 2) there is a floral/fruit transcriptome available from this species and fresh tissue as it is widely cultivated in the tropics; and 3) it exhibits a dry dehiscent fruit with complete valve separation from a ring-like persistent septum (opercular dehiscence) that resembles the silique or silicle of *Arabidopsis* (Fig. 1) [34].

## Methods

### Transcriptome analysis

We prepared transcriptomes from *Aristolochia fimbriata* (Aristolochiaceae), *Bocconia frutescens* (Papaveraceae), *Cattleya trianae* (Orchidaceae), and *Hypoxis decumbens* (Hypoxidaceae), as previously described [35–37]. Fresh inflorescence, floral tissue, and vegetative tissue from all mentioned species were ground using liquid nitrogen, and further total RNA extraction was carried out using Trizol Reagent (Invitrogen). RNAseq experiments for each species were conducted using truseq mRNA library construction kit (Illumina) and sequenced in a HiSeq 2000 instrument reading 100 bases paired end reads. Read cleaning was performed with PRINSEQ-LITE with a quality threshold of Q35, and contig assembly was computed using Trinity package following default settings. For *Aristolochia fimbriata*, contig metrics are as follows: total assembled bases: 85,608,833; total number of contigs (>101 bp): 118,941; average contig length: 719 bp; largest contig: 16,972 bp; contig N50: 1823 bp; contig GC%: 42.71% [35]. For *Bocconia frutescens*, contig metrics are as follows: total assembled bases: 149,710,500; total number of contigs (>101 bp): 211,821; average contig length: 706 b; largest contig: 17,004 b; contig N50: 1877 bp; contig GC%: 40.09 [36]. For *Cattleya trianae,* contig metrics are as follows: total assembled bases: 63,287,862 bp; total number of contigs (>101 bp): 109,708; average contig length: 576 bp; largest contig: 9,321 bp; contig N50: 1,401 bp; contig GC%: 42,73 [37]. For *Hypoxis decumbens,* contig metrics are as follows: total assembled bases: 73,787,751 bp; total number of contigs (>101 bp): 157,153; average contig length: 469 bp; largest contig: 15,554 bp; contig N50: 1,075 bp; contig GC%: 46,42 [37].

### Phylogenetic analyses

To expand sampling of *SPT/ALC* homologs, we isolated sequences using BLAST from our own generated transcriptomes from *Aristolochia fimbriata*, *Bocconia frutescens, Cattleya trianae* and *Hypoxis decumbens*. To show the phylogenetic position of the *Bocconia frutescens* homologs, we included *BofrSPT1, 2* and *3* in a matrix consisting of selected ALC and SPT from all major plant groups, expanding the sampling done by Pabón-Mora et al. [29]. Most sequences were obtained from the plant transcriptome repositories of the OneKP database (https://sites.google.com/a/ualberta.ca/onekp/) and the genome repository Phytozome (https://phytozome.jgi.doe.gov/pz/portal.html). Sequences were compiled with Bioedit (http://www.mbio.ncsu.edu/bioedit/bioedit.html) and manually edited to exclusively keep the open reading frame for all transcripts, as many sequences from transcriptomic databases include the 5′ and 3′ untranslated reading frames (UTR’s). Nucleotide sequences were subsequently aligned using the online version of MAFFT (http://mafft.cbrc.jp/alignment/software/) [38] with a gap open penalty of 3.0, offset value of 1.0, and all other default settings. The alignment was then refined by hand using Bioedit around the bHLH domain. Maximum likelihood (ML) phylogenetic analyses using the nucleotide sequences were performed with RaxML-HPC2 BlackBox [39], through the CIPRES Science Gateway [40]. Bootstrapping was performed according to the default criteria in RaxML where the bootstrapping stopped after 200–600 replicates. Trees were observed and edited using FigTree v 1.4.3. (http://tree.bio.ed.ac.uk/software/figtree/). This analysis included all 54 sequences used for motif search (see above) from angiosperms and used the 6 gymnosperm paleoSPT/ALC outgroup sequences (Additional file 1: Table S1). Newly isolated sequences from our own generated transcriptomes from *Aristolochia* *fimbriata,* *Bocconia frutescens*, *Cattleya trianae* and *Hypoxis decumbens* are available under Genbank numbers KY421362–KY421369.

### Identification of protein motifs across flowering plants

To detect reported as well as new conserved motifs, 60 complete sequences of *SPT/ALC* homologs were selected representing major seed plant lineages (25 from core eudicots, 9 from basal eudicots, 14 from monocots, 6 from basal angiosperms and 6 from gymnosperms). Sequences were permanently translated and uploaded as amino acids to the online MEME server (http://meme.nbcr.net) [41] and run with all the default options. The motifs retrieved by MEME are reported according to their statistical significance. The suite MEME finds in the given sequences the most statistically significant (low E-value) motifs first. We did the search for 20 motifs arbitrarily to search beyond the already identified motifs for SPT and ALC homologs. The E-value of a motif is based on its log likelihood ratio, width, sites, and the size of the set. The motifs identified with our dataset range from 9.3 e to 3095 (motif 1) found in all 60 input sequences, to 2.7 e–0.25 (motif 20) found in only 5 sequences (Additional file 2: Figure S2). Protein motifs provide important information for better assessing shifts in SPT/ALC proteins across seed plants, and as all motifs found are statistically significant (none of them is larger than 0.05) we decided to report them here even though more detail functional analyses are required in order to better understand how meaningful are the shifts here identified.

### Developmental series of flowers and fruits of *B. frutescens*

Inflorescences, young buds, flowers and fruits were collected in the field (voucher: Colombia, Antioquia, Medellín, Las Palmas, Envigado, sobre la via principal, Km 12 retorno No 10. May 2015, *C. Zumajo*-*Cardona and N. Pabón*-*Mora* 03, HUA) and immediately fixed in formaldehyde-acetic acid–ethanol (FAA; 3.7% formaldehyde: 5% glacial acetic acid: 50% ethanol). For light microscopy, fixed material was manually dehydrated through an alcohol-histochoice series, and embedded in Paraplast X-tra (Fisher Healthcare, Houston, Texas, USA). The samples were sectioned at 10–20 µm with an AO Spencer 820 (GMI Inc. Minnesota, USA) rotary microtome. Sections were stained with Johansen`s safranin, to identify lignification and presence of cuticle, and 0.5% Astra Blue [42] and mounted in Permount (Fisher Scientific, Pittsburgh, Pennsylvania, USA). Sections were viewed and digitally photographed with a Zeiss Axioplan compound microscope equipped with a Nikon DXM1200C digital camera with ACT − 1 software. Different stages in flower and fruit development of *B. frutescens* were described using stages already identified in this species and other Papaveraceae as a Ref. [36, 44, 45]. Late fruit developmental stages were photographed and included in Fig. 1. In addition, a comparative drawing with respect to the Arabidopsis fruit was done based on fresh material and it corresponds to Fig. 1b.

### Expression analyses by *In Situ* Hybridization

Inflorescences with flowers at different stages as well as mature carpels and immature fruits of *B. frutescens* were collected in the field (voucher: Colombia, Antioquia, Medellín, Las Palmas, Envigado, sobre la via principal, Km 12 retorno No 10. May 2015, *C. Zumajo*-*Cardona* and *N. Pabón*-*Mora* 03, HUA) and fixed in freshly prepared, cold FAA. After a 4-h incubation, samples were dehydrated in an ethanol series and then transferred to fresh Paraplast and stored at 4 °C until use. Samples were sectioned with a microtome at 10 µm. Samples were sectioned on a Microm HM3555 rotary microtome. DNA templates for RNA probe synthesis were obtained by PCR amplification of 312–412 bp fragments. To ensure specificity, the probe templates were designed to amplify the 3′ sequence flanking the bHLH domain (Additional file 3: Figure S1; Additional file 4: Table S2). Because of the high percentage of similarity in the sequences, we were not able to design specific probes that recognized *BofrSPT1* from *BofrSPT2*; thus, there is a single *BofrSPT1/2* probe, and a different probe for *BofrSPT3* (Additional file 3: Figure S1). Fragments were cleaned using QIAquick PCR purification Kit (Qiagen, Valencia, CA, USA). Digoxigenin labeled RNA probes were prepared using T7 polymerase (Roche, Switzerland), murine RNAse inhibitor (New England Biolabs, Ipswich, MA, USA), and RNA labeling-mix (Roche, Switzerland) according to each manufacturers protocol. RNA in situ hybridization was performed according to Ambrose et al. [45] and Ferrándiz et al. [46], optimized to hybridize overnight at 55 °C. *In situ* hybridized sections were subsequently dehydrated and permanently mounted in Permount (Fisher, Waltham, MA, USA). All sections were digitally photographed using a Zeiss Axioplan microscope equipped with a Nikon DXM1200C digital camera.

## Results

### *ALCATRAZ/SPATULA* gene evolution

**Fig. 2:**
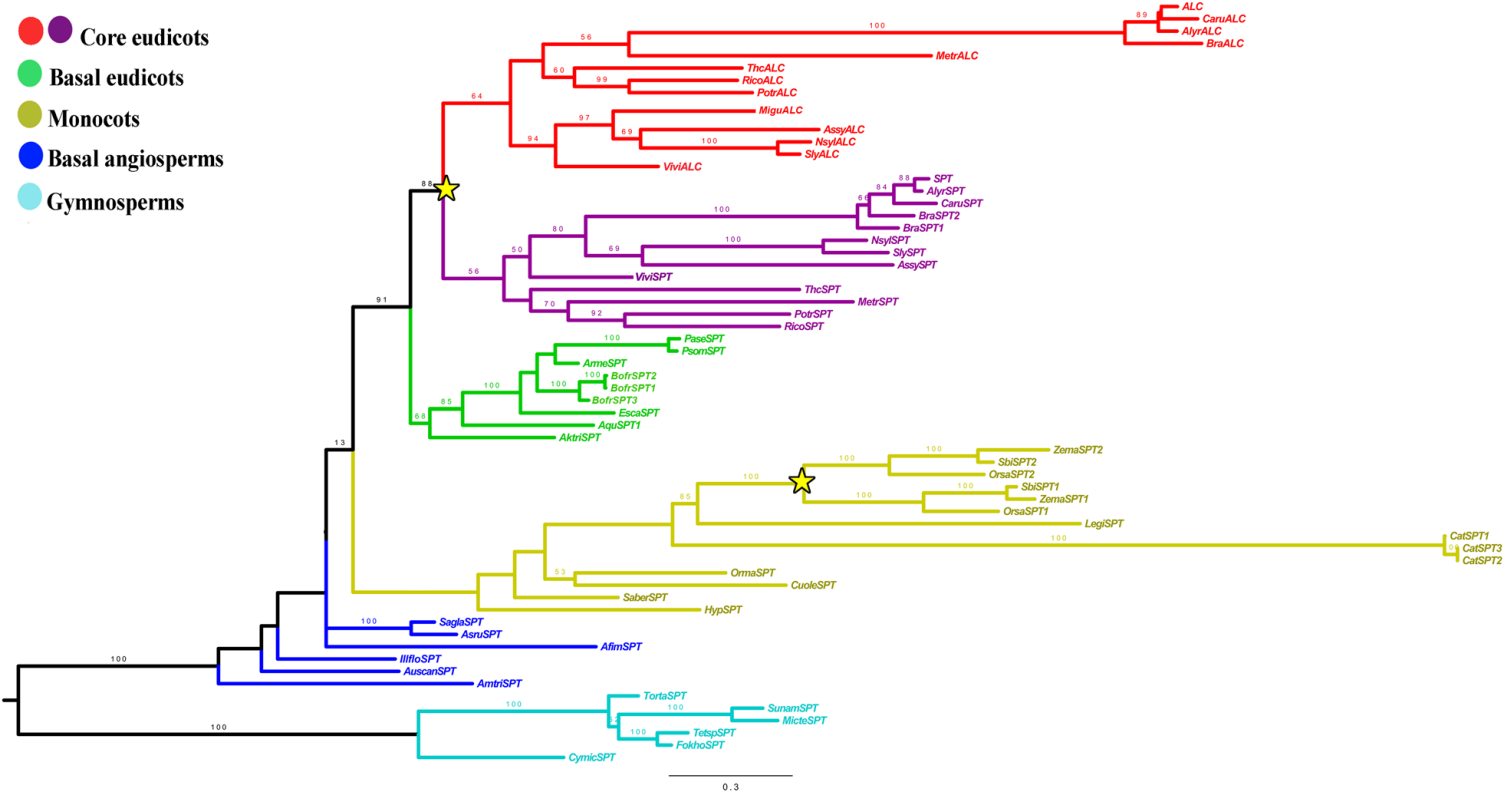
Maximum likelihood tree of *SPATULA*/*ALCATRAZ* genes in seed plants. The topology recovers that of Pabón-Mora et al. [29]. All Bootstrap (BS) values are placed at nodes. *Yellow stars* indicate two large scale duplication events, the first one in the Poaceae and the second one coincides with the core eudicots giving rise to the *SPT* and *ALC* clades

To reconstruct the *SPT/ALC* gene lineage evolution, we included 60 sequences from all major seed plant groups. Unlike our previous analysis that used exclusively the bHLH domain [29], here we have included the complete coding sequences of all homologs. The resulting topology, however, shows the same two duplication events we previously reported (Fig. 2). One duplication event correlates with the diversification of the Poaceae, with a very high support (100 BS bootstrap value) and another duplication (BS 88), results in the SPT (BS 56) and ALC (BS 64) clades in core eudicots.

**Fig. 3:**
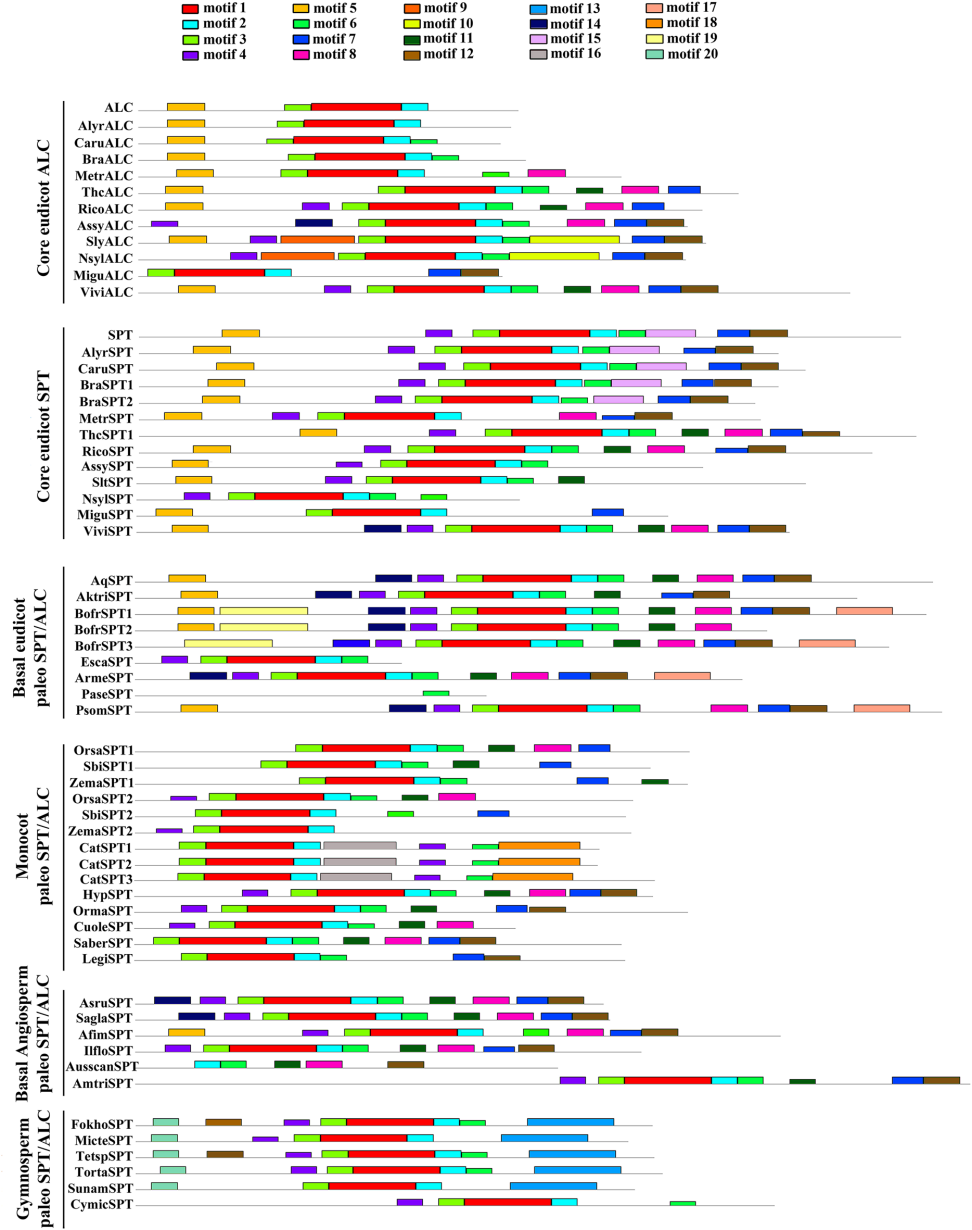
Conserved motifs of SPT/ALC proteins across seed plants identified through a MEME analysis. Each motif is represented by a colored box numbered at the *top*. The *black lines* represent unique sequences. The bHLH domain (here represented by motifs 1, 2 and 3) is highly conserved across seed plant sequences. Other conserved motifs include the acidic domain (motif 4), the amphipathic helix (motif 5) and the β strand (motif 6). *Scale bar* indicates number of amino acids (AA). Names to the *left* indicate the clades to which the sequences belong to according to Fig. 2

**Fig. 4:**
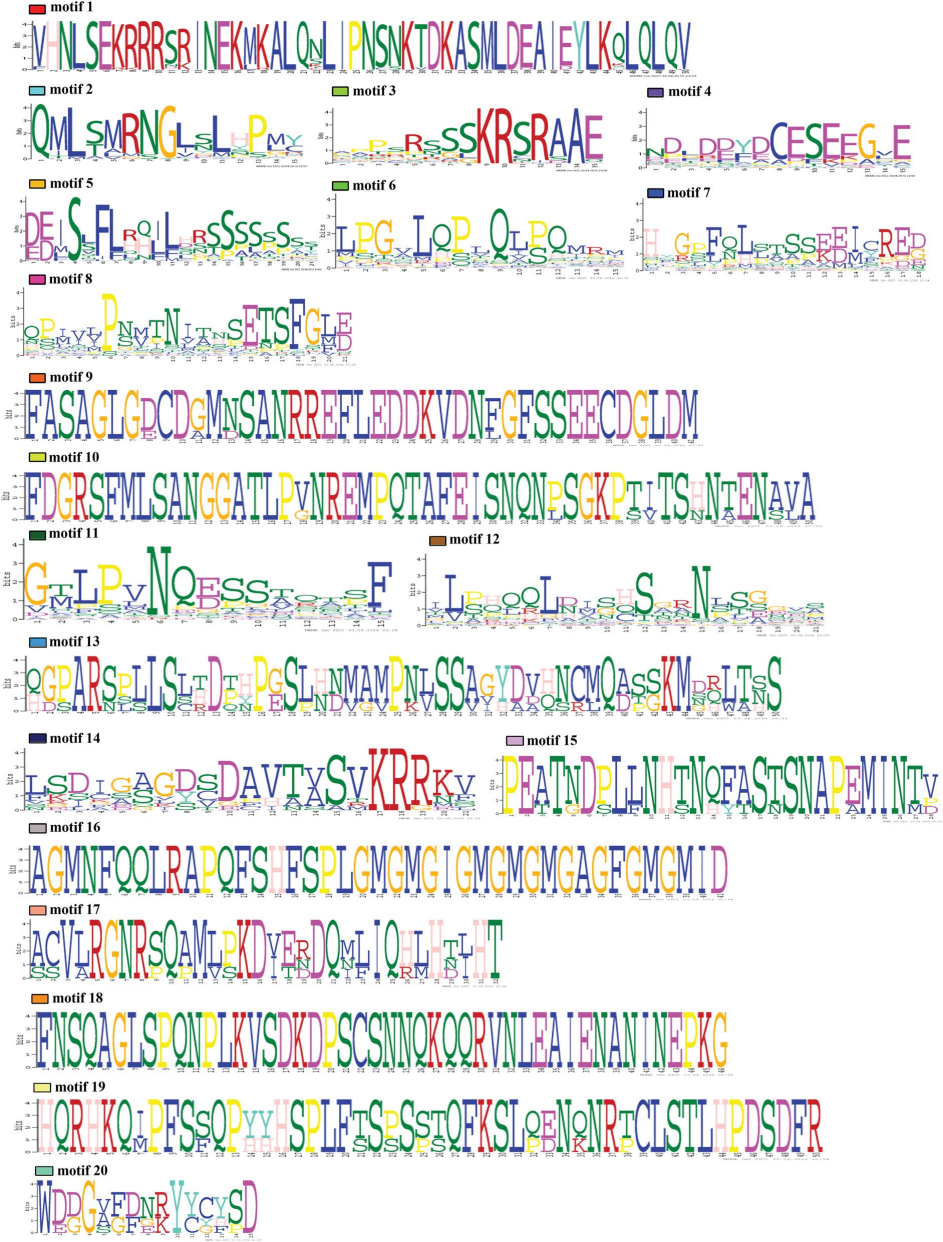
Sequences of the conserved motifs detected by the MEME analysis on the SPT/ALC homologs across seed plants. *Letter size* denotes the degree of conservation of each amino acid

Our MEME analysis resulted in the identification of conserved protein motifs across homologous sequences in seed plants as well as more specific motifs only present in some taxa (Figs. 3, 4). We found that the bHLH domain is highly conserved in all seed plant sequences, corresponding to previously identified motifs 1, 2 and 3 (Figs. 3, 4) [21, 29]. In the 5′ flank of the bHLH domain, we identified motif 4, corresponding to the acidic domain [21, 29] DDY/FDCESEEGVE, present in 42 sequences out of the 60 sampled. Motif 4 is lacking from all Brassicaceae ALC homologs, as well as in many monocot paleoSPT/ALC. Motif 5, D/EE/DI/MSL/FRSSSSSSSS, corresponds partly to the amphipathic helix (DELSSF/IRQI/VL) reported by Groszmann et al. [21]. It is rich in serine and is present in all eudicot SPT and ALC protein sequences, whereas it is lacking in monocots, basal angiosperms or gymnosperms. Motif 6 is the β strand [sensu Grozsmann et al. 2011]; (LPGxLQPxQLPQ); it is found right after the 3′ end of the bHLH domain and is conserved in most angiosperm homologs sampled except in ALC, some monocot proteins (ZemaSPT2, SbiSPT2) and some gymnosperm sequences (MicteSPT, SunamSPT and CymicSPT). Motif 7 (HxGP/SFQLS/LT/ASSEEICRED) is located toward the end of almost all protein sequences, except in the Brassicaceae ALC copies, *Medicago truncatula* ALC (MtrALC) and the gymnosperm homologs. Motif 8 seems to be present only in angiosperms SPT/ALC homologs, but is apparently lost in Brassicales as neither ALC nor SPT Brassicaceae proteins have it. Motifs 9 and 10 are exclusive to ALC Solanaceae homologs. Motif 11 G/VT/MLPV/LNQE/DSST/AxxxF is present in most non-core eudicot proteins, but it is absent in Brassicaceae SPT and ALC homologs. Motif 12 is at the C-terminus of most proteins, except for two gymnosperm sequences (FokhoSPT and TetspSPT) that have motif 12 at the N-terminus. Motifs 13 and 20 are exclusive of gymnosperm homologs. Motif 14 (DAVTV/ASVKRRKV/F) is present in basal eudicot copies, the Chloranthales homologs, and the *Vitis vinifera SPT* copy (ViviSPT). Similarly, motif 17 is characteristic of most basal eudicot SPT/ALC homologs, including two of the three *Bocconia frutescens* SPT/ALC proteins. Motif 10 is exclusive of the Solanaceae ALC orthologs, whereas motif 15 is exclusive of the Brassicaceae SPT orthologs. Finally, motifs 16 and 18 are exclusive to the C-terminus of the three *Cattleya trianae* (Orchidaceae) SPT/ALC homologs (Figs. 3, 4).

### Expression of paleo*SPT/ALC B. frutescens* orthologs (*BofrSPT1*/2/3)

**Fig. 5:**
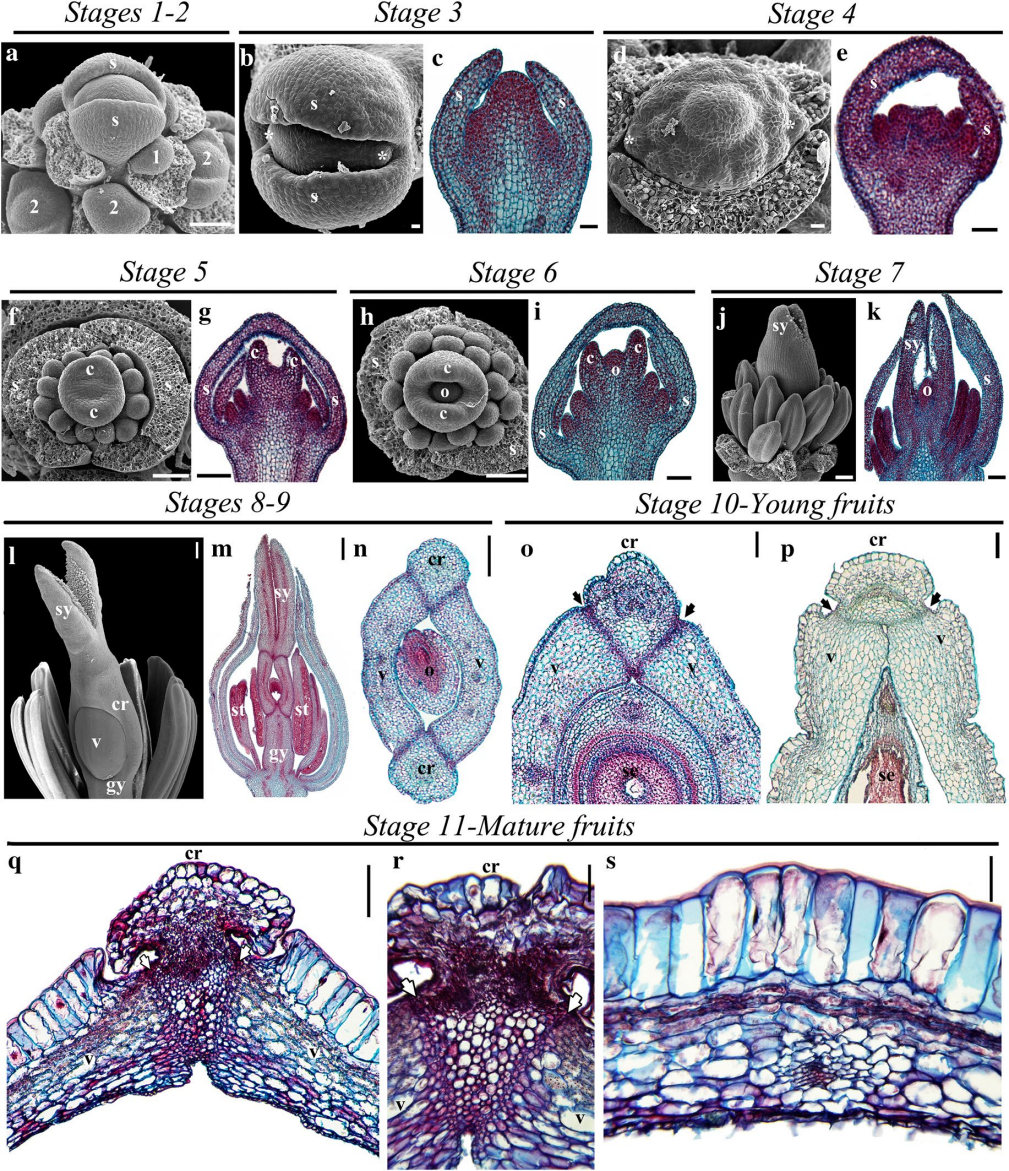
Flower and fruit development stages of *Bocconia frutescens* shown in SEM and anatomical sections. **a** Inflorescence apex with floral primordia in stage 1 and floral buds during sepal initiation in stage 2. **b**, **c** Floral buds in the stage 3 during stamen initiation in the second whorl. **d**, **e** Floral buds in the stage 4 during stamen initiation in the next three inner whorls. **f**, **g** Floral buds in stage 5 during carpel initiation. **h**, **i** Floral buds in stage 6 during when the ovule is formed. **j**, **k** Floral buds in stage 7 during stigma elongation. **l–n** Floral buds in stages 8 and 9 during carpel proximo-distal differentiation. Note the formation of the gynophore, the valves and the commissural ring. **o–s** Cross sections of *B. frutescens* fruits. **o–p** Young fruits (stage 10) with close up to the dehiscence zone in a young fruit (3 mm diameter), right after anthesis in the center (**o**) and the tip (**p**). **q–s** Mature fruits at stage 11 with close up to the dehiscence zone, the commissural ring and its main vascular bundle (**q**, **r**) and fruit wall (**s**) of a more mature fruit (6 mm diameter) of *B. frutescens.* 1: stage 1, 2: stage 2, *c* carpel, *cr* commissural ring, *gy* gynoecium, *o* ovule, *s* sepal, *st* stamen, *sy* stigma, *v* valve, *: first pair of stamens that appear in the place of petals. Arrows indicate the dehiscence zone. *Scale bars*: 10 μm (**b**, **d**), 40 μm (**r**, **s**) 50 μm (**c**, **e**, **q**), 100 μm (**a**, **f**, **g**, **h**, **i**, **j**, **k**, **o**, **p**), 200 μm (**l**), 250 μm (**n**, **o**), 500 μm (**m**)

*Bocconia frutescens* L., has determinate inflorescences with numerous flowers, where partial inflorescences are formed by 2–3 apetalous flowers formed by two sepals, a single whorl of homeotic stamens replacing petals, two–three whorls of true stamens and finally, a bicarpellate gynoecium with papillose exerted stigmas [36]. Flowers develop basipetally with the terminal flower always bigger. In order to properly describe the molecular genetics of fruit and flower development in *B. frutescens*, we performed developmental analyses by scanning electron and light microscopy. Similar to other floral developmental studies, particularly in the Papaveraceae [43, 44], we have defined 11 stages of floral and fruit development based on the following landmarks (Table 1; Fig. 5). Stage 1—the floral meristem can first be distinguished (Fig. 5a). Stage 2—the two sepal primordia initiate (Fig. 5a). Stage 3—usually corresponding to petal initiation, hereby replaced by the first whorl of homeotic stamens (Fig. 5b, c). Stage 4—the next two to three staminal whorls are formed (Fig. 5d, e). Stage 5—the initiation of the bicarpellate gynoecium closing the floral meristem (Fig. 5f, g). Stage 6- the two carpels overtop the single ovule (Fig. 5h, i) and Stage 7—the apical most distal regions of the gynoecium including the style and the stigma differentiate (Fig. 5i, k). During Stage 8 the gynoecium in *B. frutescens* differentiates a medial–lateral plane of two valves derived from the two carpels, each with a central midvein, separated by a persistent commissural ring-like tissue, also irrigated by two massive vascular traces (Fig. 5l–n). It also differentiates three main proximo-distal zones including a gynophore, an ovary and a short style with two massive vascularized stigmas (Fig. 5l–n). The ovary wall is formed by 12 layers including both the outer and inner epidermis in pre-anthesis (Fig. 5n) and remains the same in anthesis (Stage 9).

**Table 1:** Developmental landmarks for each stage identified during flower and fruit development

Stage	Developmental landmarks
Stage 1	The floral meristem can first be distinguished (Fig. 5a)
Stage 2	The two sepal primordia initiate (Fig. 5a)
Stage 3	Initiation of the first whorl of homeotic stamens (Fig. 5b, c)
Stage 4	Formation of the next two to three staminal whorls (Fig. 5d, e)
Stage 5	Initiation of the bicarpellate gynoecium closing the floral meristem (Fig. 5f, g)
Stage 6	Overtopping of the two carpels around the single ovule (Fig. 5h, i)
Stage 7	Differentiation of the style and the stigma (Fig. 5i, k)
Stage 8	Medial–lateral plane differentiation in the carpel. Two valves are distinguished, each with a central midvein, separated by a persistent commissural ring-like tissue, also irrigated by two massive vascular traces (Fig. 5l–n)
	Differentiation of the proximo-distal zones including a gynophore, an ovary and a short style with two massive vascularized stigmas (Fig. 5l–n)
Stage 9	Anthesis. Formation of up to 12 layers in the ovary wall including both the outer and inner epidermis
Stage 10	Young fruits. Expansion of the valves by both anticlinal and limited periclinal cell division reaching up to 15 layers in the fruit wall (Fig. 5o, p)
	Expansion of the commissural ring outwards developing a larger central vascular bundle surrounded abaxially and adaxially by collenchyma (Fig. 5o, p)
Stage 11	Mature fruits. Radial elongation of the outer epidermis accompanied by tangential elongation of the hypodermal cell layers in the mesoderm (Fig. 5q–s)
	Flattening of the two inner-most cell layers in the endoderm in the periphery of the dehiscence zone where they expand and become sclerenchymatic (Fig. 5q–s)
	Formation of the dehiscence zone by 2–3 layers of smaller cells in the limits between the commissural ring and the fruit valves (Fig. 5q–s)
Stage 12	Opercular dehiscence, which occurs between the valves and the persistent ring-like tissue and the single seed remains attached to the base of the ring through the funicle, exposing a fleshy red aril that may be a bird attractant tissue for seed dispersal (Fig. 1c, d)

During fruit development, after anthesis, two additional stages were identified: Stage 10 is defined by young fruits, when the valves expand by both anticlinal and limited periclinal cell division reaching up to 15 layers in the fruit wall (Fig. 5o, p). The commissural ring also expands outwards and develops a larger central vascular bundle surrounded abaxially and adaxially by collenchyma (Fig. 5o, p). Stage 11 is defined by mature fruits, when the outer epidermis continues to enlarge into rectangular radially elongated cells and while the hypodermal cell layers in the mesoderm start to tangentially elongate the cells toward the inside of the vascular bundles begin cell expansion (Fig. 5q–s). The two inner-most cell layers in the endoderm are flattened, except in the periphery of the dehiscence zone where they expand and become sclerenchymatic (Fig. 5q–s). The dehiscence zone is formed by 2–3 layers of smaller cells in the limits between the commissural ring and the fruit valves. No adjacent layers of lignified cells can be observed at any developmental stage (Fig. 5q–s). The opercular dehiscence marks Stage 12, which occurs between the valves and the persistent ring-like tissue and the single seed remains attached to the base of the ring through the funicle, exposing a fleshy red aril that may be a bird attractant tissue for seed dispersal (Fig. 1c, d).

As there are superficial similarities between the dry dehiscent fruit with complete valve separation from a ring-like persistent replum (commissural ring) by opercular dehiscence in *Bocconia* and that of the silique of *Arabidopsis*, the comparison between the genetic mechanisms controlling fruit dehiscence in these distantly related taxa becomes even more relevant (Fig. 1). In this context, the study of expression of *paleoSPT/ALC* genes in *B. frutescens* serves as a starting point to better assess the role of *SPT/ALC* genes prior to the core eudicot duplication and thus the functional evolution of the *SPT/ALC* gene lineage. Using in situ hybridization, we evaluated the expression patterns of *paleoSPT/ALC* copies found in the transcriptome of *B. frutescens* during flower and fruit development. Although we found three *paleoSPT*/*ALC* genes due to sequence similarity, we were not able to design different probes for *BofrSPT1* and *BofrSPT2*, in consequence, we used a single probe to detect *BofrSPT1/BofrSPT2* and a second gene specific probe for *BofrSPT3* (Additional file 3: Figure S1).

**Fig. 6:**
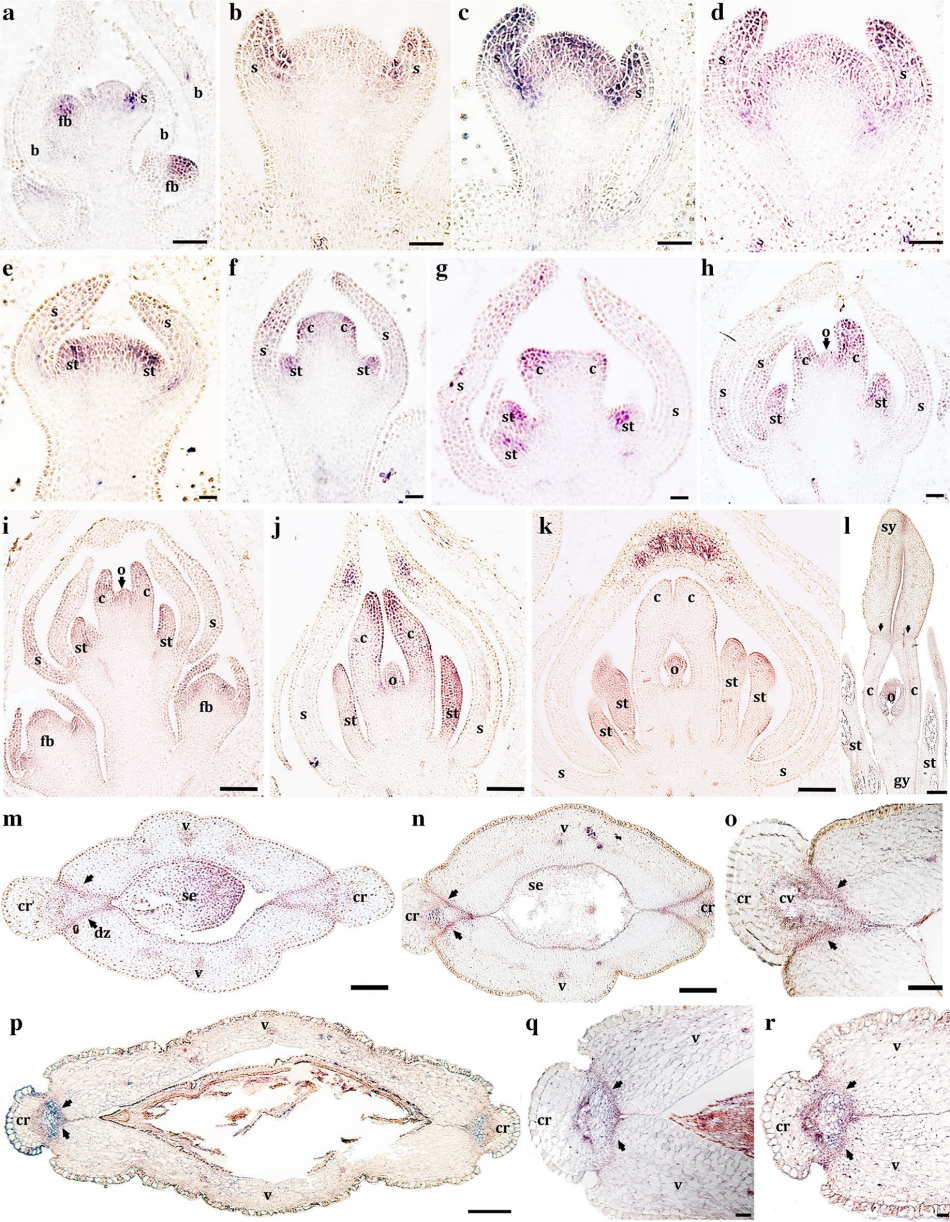
Expression of *BofrSPT1*/2 using *In situ* mRNA hybridization. **a** Inflorescence apex with floral buds in stages 1 and 2. **b–i** Floral stages 2–6. **J**, **k** Floral stage 7. **l** Floral stage 8. **m–r** Young fruits in cross section at the mid-level (**m**–**o**) and at the tip (**p**–**r**). *Black arrowheads* point to the dehiscence zones. *b* bract, *fb* floral bud, *s* sepal, *st* stamen, *c* carpel, *o* ovule, *sy* stigmata, *gy* gynoecium, *v* valve, *cr* commissural ring, *cv* central vascular bundle in the commissural ring, *dz* dehiscence zone, *se* seed. *Scale bars*: 100 μm (**a**–**g**, **h**), 0.2 mm (**i**, **k**, **m**, **n**), 0.1 mm (**l**, **o**, **q**, **r**), 0.5 mm (**p**)

**Fig. 7:**
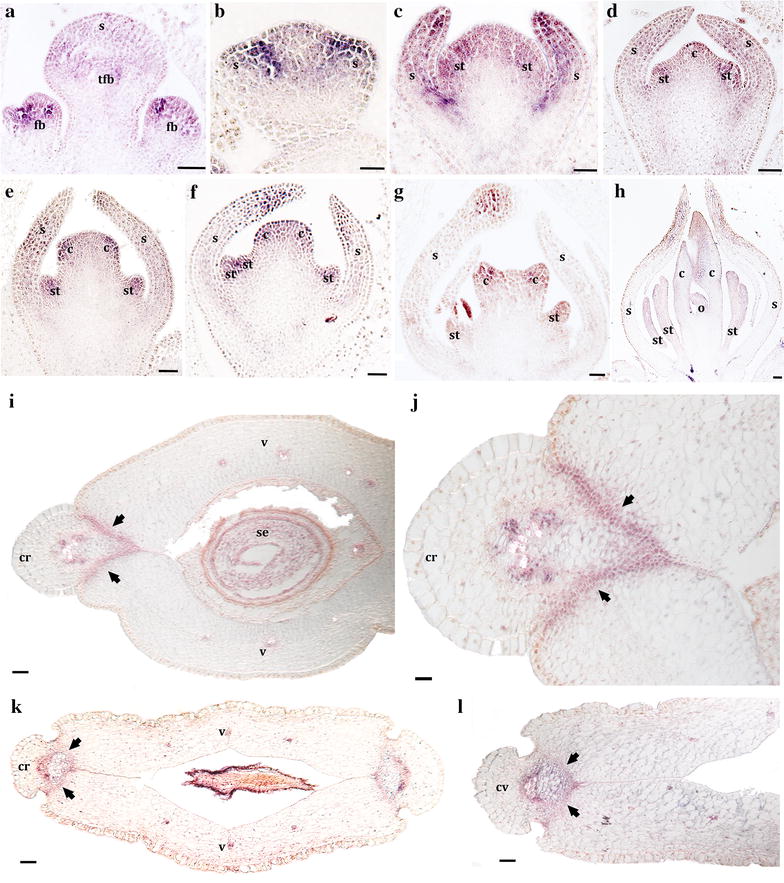
Expression analysis of *BofrSPT3* using *In situ* hybridization. **a** Inflorescence apex with floral buds in stages 1 and 2. **b–h** Floral stages 2–7. **i–l** Young fruits in cross section at the mid-level (**i**, **j**) and at the tip (**k**, **l**). *Black arrows* point to the dehiscence zone. *c* carpel, *cv* central vascular bundle in the commissural ring, *cr* commissural ring, *fb* floral bud, *s* sepal, *st* stamen, *tfb* terminal floral bud, *v* valve. *Scale bars*: 50 μm (**j**), 100 μm (**a**–**h**), 0.1 mm (**i**, **k**, **l**)

Our results show that *BofrSPT1/SPT2* and *BofrSPT3* have similar expression patterns early in flower development. *BofrSPT1/2/3* are turned on in the youngest floral meristem in stages 1 and 2, particularly strong in the adaxial surface of the sepals and in the limits between the sepals and the rest of the developing floral primordia (Figs. 6a, b; 7a, b). All three copies are expressed during stages 3 and 4 in the growing tips of the sepals, in the developing stamen primordia, and in the center of the floral meristem prior to carpel initiation (Figs. 6d, e; 7c). During stages 5 and 6, all copies expand their expression to the elongating carpel primordia and to the ovule and are continuously expressed in the distal-most portion of the stamens and restricted only to the tip of the sepals (Figs. 6f–i; 7e–g). During stage 7, *BofrSPT1/2/3* are expressed in the growing tips of the two carpels that fuse to each other enclosing the single ovule (Figs. 6j, k; 7h). Later during carpel development until anthesis (stages 8 and 9), the expression patterns of *BofrSPT1/2* and *BofrSPT3* begin to diverge. During carpel elongation and differentiation, *BofrSPT1/2* are expressed in the elongating stigmas, particularly toward the adaxial epidermis of each massive stigma (Fig. 6l) but *BofrSPT3* is not. Some expression of *BofrSPT1/2* is detected in the junction between the ring-like structure derived from the commissural tissue in between the carpels and the upper stigmas (Fig. 6l), but *BofrSPT3* expression is not detected in these same areas. During the transition to fruit development (stages 10–11), *BofrSPT1*/2/3 are expressed in the 3–4 cell layers between the valves and the commissural ring that will form the dehiscence zone in the mature fruits (Figs. 6m–o; 7i, j). *BofrSPT3* is also expressed in the phloem cells in the vascular bundle directly in contact with the xylem cells forming the commissural vascular bundle (Fig. 7i, j). During late fruit development prior to the dehiscence (stage 11), all three paralogs are expressed in the dehiscence zone that becomes compressed as both the valves and the commissural ring thicken (Figs. 6p–r; 7k, l).

## Discussion

Our phylogenetic analysis confirms that a major duplication event in the *SPT/ALC* gene lineage, occurred at the base of the core eudicots and coincides with the γ whole genome duplication (WGD) 130 Mya [9, 29]. This finding contradicts previous reports that point to a Brassicaceae-specific duplication of this gene lineage in the Brassicaceae, at the β WGD event, 40–70 Mya [21, 47]. Understanding how sequences evolve and how their changes underpin diversity is one of the major questions in evolutionary developmental studies. In the absence of functional data is not possible to tease apart differences in function due to protein interactions, the expression domains, and interactions in trans [27, 48–51, 52]. However, in the absence of functional data, we can provide a framework for future studies based on sequence analyses and expression analyses.

One of our goals was to determine how conserved are the protein domains in these sequences across seed plants as this can indicate conservation or changes in protein–protein interactions of SPT, ALC, and/or paleoSPT/ALC, with the caveat that presence/absence of motifs is often not sufficient to explain functional specificity [50]. Our expanded dataset has allowed us to confirm that the bHLH domain and the 3′ ß-strand (LQLQVQ; motif 6) are highly conserved in all analyzed sequences (Figs. 3, 4). This suggests that the function in conferring specificity typically assigned to the basic region, as well as the ability to form homo- or heterodimers of the bHLH region, are both maintained in ALC, SPT as well as in paleoSPT/ALC proteins (Fig. 3) [13, 29, 53, 54]. Because both the bHLH domain and the flanking regions are known to be necessary and sufficient for SPT homodimerization and the heterodimerization with other bHLH proteins, it is possible that some SPT/ALC proteins in other flowering plants could share some common partners when compared to those identified for SPT and ALC. A persistent motif in SPT and ALC clades [13, 21] and also in the basal eudicots proteins is the amphipathic helix in motif 5, located near the N- terminus of the protein (Figs. 3, 4). This amphipathic helix is required for full complementation of *spt* mutants in Arabidopsis, and it is thought to function in recruiting co-activators [13]. Our data show that motif 5 is only present in eudicots, and although core eudicot *SPT* functional data is scarce, at least *SlySPT* (the *Solanum lycopersicum* SPT homolog) can fully complement and restore *spt* mutants in *Arabidopsis,* although this does not necessarily mean their functions are conserved [13]. Because motif 5 is lacking outside eudicots it is possible that functions reported for the amphipathic helix are likely not plesiomorphic in the lineage.

**Fig. 8:**
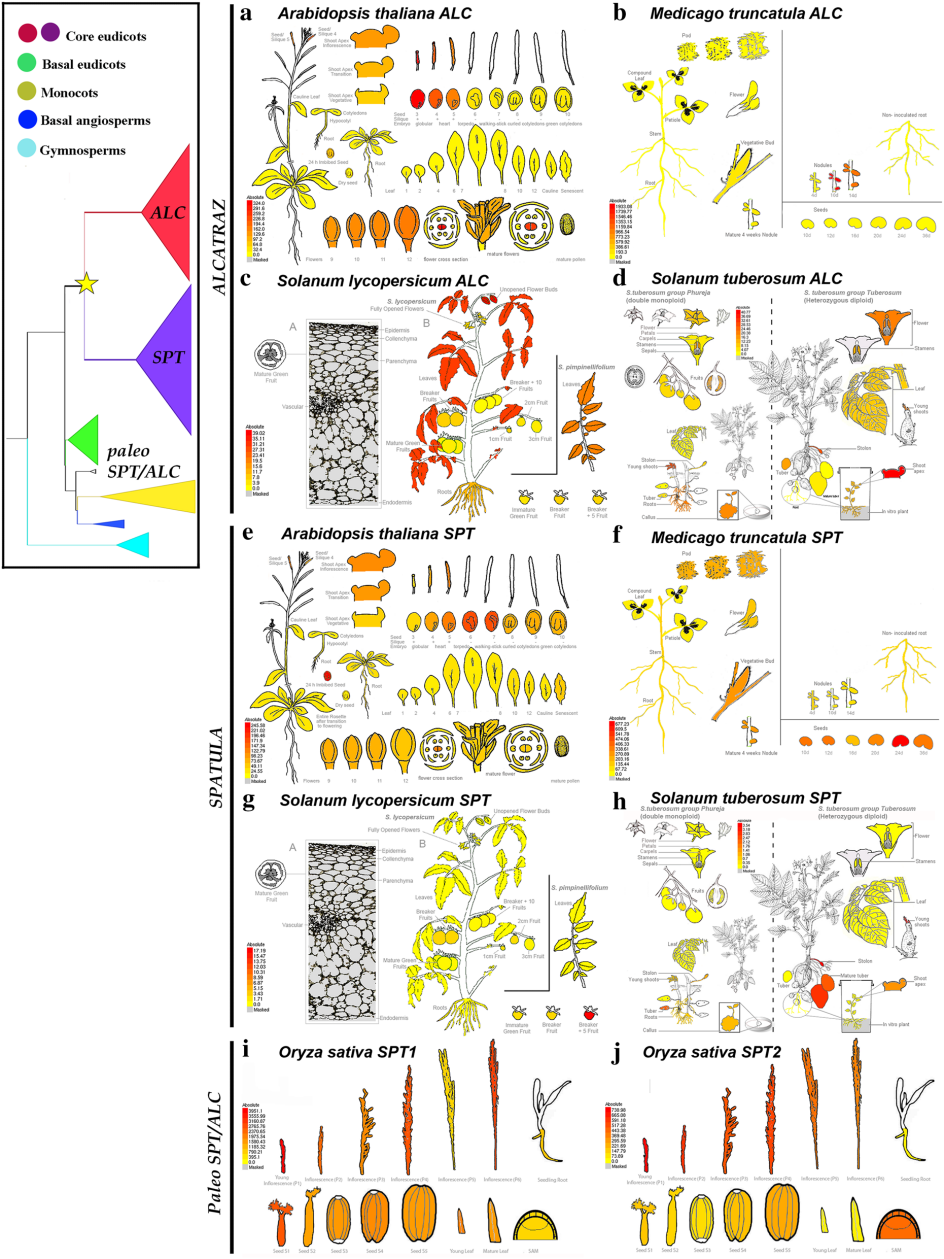
Expression patterns of selected *ALCATRAZ/SPATULA* homologs from selected model species. **a–d** Expression of *ALCATRAZ* homologs from core eudicots including *Arabidopsis thaliana* (At5g67110), *Medicago truncatula* (Medtr1g019240), *Solanum lycopersicum* (Solyc04g078690), *Solanum tuberosum* (PGSC003DMT400020534). **e–h** Expression of *SPATULA* homologs from the same core eudicots, *A. thaliana* (At4g36930), *M. truncatula* (Medtr5g017040), *S. lycopersicum* (Solyc02g093280), and *S. tuberosum* (PGSC003DMP400007151). **i–j** Expression of *paleoALC*/*SPT* from *Oryza sativa* (monocot), OrsaSPT1 (LOC_Os06g06900) and OrsaSPT2 (LOC_Os02g56140). Taken from the eFP browser (http://bar.utoronto.ca/efp/cgi-bin/efpWeb.cgi)

The acidic domain (motif 4) was found in *SPT*, *ALC* and paleo*SPT/ALC* but not in Brassicaceae *ALC* sequences and SPT1 orthologs in monocots [21, 29]. Thus, the acidic domain is present in gymnosperms, basal angiosperms, SPT2 orthologs in monocots, and basal eudicots. The acidic domain is key to restoring *spt Arabidopsis* mutants, and it is absolutely required for *SPT* function [13]. A close inspection of the rice paralogs indicates that OrsaSPT1 lacks the acidic domain but possesses motif 7, which is rich in Phenylalanine (F), Leucine (L), Serine (S) and Glutamic Acid (E) similar to an acidic motif. Moreover, according to the eFP Browser, *OrsaSPT1* is co-expressed with *OrsaSPT2*, pointing to an interesting system to test functional evolution of close paralogs and shifts in protein folding and putative partners (Fig. 8). Altogether, this suggests that sequences with the acidic domain across seed plants could have functional resemblance and a closer set of interactions to SPT compared to ALC.

It had been hypothesized that the *SPT/ALC* genes, in comparison with their closely related bHLH *PIF 3/4/5* genes, lost the 5′ APB domain, which is a negative regulator of shade responses [15]. Such evolutionary loss is a prerequisite for the recruitment of *SPT* homologs in carpel development, in a light-independent manner [15], and our analyses confirm that there are no remnants of an APB domain in any of the seed plant *SPT/ALC* sequences. Interestingly, motifs 8 and 14 are only present in angiosperms and lacking in gymnosperm homologs, whereas motifs 13 and 20 are exclusive to gymnosperms. However, the *Amborella trichopoda AtriSPT* sequence lacks both motifs 8 and 14, suggesting that there are no synapomorphic motifs for all angiosperm sequences likely responsible for new capabilities in carpel development.

Our analysis corroborates previous findings that all model core eudicot plants including Arabidopsis, tomato, potato and Medicago have orthologs to SPT and to ALC as the duplication event occurred prior to their diversification [29]. This observation poses a new question regarding conservation in their expression patterns after the duplication as well as the expression patterns and putative roles of pre-duplication genes. In Arabidopsis, ALC and SPT are co-expressed in the leaf margin, medial ridge of the gynoecium by stage 8, in the valve margin from stage 9 and in the septum outgrowth and transmitting tract as well as in the stigma during stage 11 and in the ovules [16, 19–21, 48]. During late fruit development, expression of both *SPT* and *ALC* also overlaps in the dehiscence zone of the fruit [13, 16, 19–21]. However, in addition to these organs, *ALC* is expressed in the petal margins, the connective of developing anthers, nectaries, and the pedicel-stem abscission zone [20, 21]. On the other hand, *SPT* is expressed during early embryogenesis, particularly in the root meristem and the procambium [16]. Coexpression of both paralogs is consistent with their conserved role during carpel development and in the specification of the non-lignified layer of the dehiscence zone [20, 21]. Nevertheless, only *SPT* can fully complement *alc* mutants, whereas *ALC* can only partly complement *spt* mutants, likely because of both the shortening, and the loss of putative ancestral motifs of *ALC* orthologs in Brassicaceae (Figs. 2, 3) [21]. Sub-functionalization has been posed as the functional evolutionary scenario after duplication, as *SPT* has a more fundamental role during early gynoecium fusion, elongation and development, with direct *HECATE1,2,3* partners, whereas *ALC* has a more prominent role in the differentiation of the separation (non-lignified) layer in the dehiscence zone [20, 21, 55, 56]. *SPT* also plays important roles in specifying valve margin differentiation and dehiscence zone formation later in development, as it is a direct target of *INDEHISCENT* (IND) another bHLH gene closely related with *HEC3* which promotes the differentiation of both the lignified and the non-lignified layers [22].

Broad expression patterns for both paralogs are uncommon in other core eudicots (Fig. 8). In *Medicago truncatula* (another rosid like *Arabidopsis*), *MetrALC* has only been detected in the root nodules, whereas *MetrSPT* is expressed in the vegetative meristem and during fruit and seed development. In *M. truncatula*, for instance, it is well known that *MetrSHP,* the most important positive regulator upstream of ALC and IND according to the model, has conserved roles in establishing the dehiscence zone and controls the coiled fruit shape [56]. According to the expression patterns found in the public databases (eFP Browser), and considering upstream regulators are conserved, it is likely that it is the *MetrSPT* and not the *MetrALC* paralog playing important roles during flower and fruit development. Similar cases of sub- or neofunctionalization are likely to be occurring in Asterids. Also in the eFP Browser, in tomato (*Solanum lycopersicum*), while *SlyALC* is expressed in leaves and to some extent in flowers, *SlySPT* is mostly restricted to later developmental stages of fruit maturation (Fig. 8). In potato (*S. tuberosum*), while *StuALC* is expressed in roots, young shoots and tubers, sepals, petals, stamens and fruits, *StuSPT* is mostly restricted to tuber development (Fig. 8). Nonetheless, a functional scenario has so far been incomplete since there is no expression data available for pre-duplication genes that allow the assessment of the plesiomorphic role of the gene lineage.

Here, we present the first expression analysis of *paleoSPT/ALC* genes in the basal eudicot *Bocconia frutescens* (Papaveraceae). The study of expression patterns during flower and fruit development in a basal eudicot, placed before the duplication event, allows us to propose hypotheses in terms of the functional evolution of the *SPT/ALC* gene lineage. In *B. frutescens*, *paleoSPT/ALC* copies are expressed throughout the early floral meristem (stage 1), in the sepal primordia and quickly after sepal initiation, their expression delimits the boundaries between sepals and the rest of the floral primordia where all remaining organs will form (stage 2) (Figs. 6a, b; 7a, b). During early and mid-flower developmental stages (stages 3–6), *paleoSPT/ALC* genes are expressed in the growing tips of stamens and carpels (Figs. 6c–i, 7c–g). Importantly, *BofrSPT1,2,3* genes are expressed in the adaxial domain of the postgenitally fused carpels and in the growing ovule, during stages 7 and 8 (Figs. 6j–l; 7h). Our results show broader expression patterns during early floral organ specification, compared to that reported for *SPT* and *ALC* in *Arabidopsis*, but point to ancestral roles in eudicot *paleoSPT/ALC* homologs in early gynoecium patterning including the formation of the septum, the stigma and the style.

In addition to the expression recorded during flower development, all three *BofrSPT1,2,3* copies remain turned on during the gynoecium to fruit transition to the smaller cells marking the dehiscence zone present between the commissural ring and the valves (Figs. 6, 7). As dehiscence is opercular, the dehiscence zone as well as *BofrSPT1,2,3* expression is present from the proximal to the distal-most portions of the ovary (Figs. 1, 6, 7). This is exactly the same as the expression patterns detected for both *SPT* and *ALC* in the separation layer of the dehiscence zone during fruit maturation in *Arabidopsis* [20, 21, 56], suggesting that a role in specifying the dehiscence zone was already present in the pre-duplication *paleoSPT/ALC* genes and it is likely conserved in all early diverging angiosperms. It is important to highlight that the *B. frutescens* opercular capsule does not form a sclerenchymatic cell layer adjacent to the smaller celled separation layer, between the commissural ring and the valves. This suggests that the role of *paleoSPT/ALC* genes in delimiting the separation layer occurs independently of the formation of a lignified layer or the maintenance of *INDEHISCENT*/*HECATE3* functions in the dehiscence zone [22].

Teasing apart the evolution of the fruit developmental network will only be possible with the knockouts of each gene in the network from diverse basal eudicot species. However, our expression data supports the idea of a conserved fruit developmental genetic network in basal eudicots like *B. frutescens*, even in the absence of strict orthologs of *FUL* and *SHP* [29]. Both the *APETALA1*/*FRUITFULL* and the *AGAMOUS*/*SHATTERPROOF* gene lineage have duplicated extensively in core eudicots, basal eudicots and monocots independently, making it extremely difficult to tease out new plesiomorphic roles in plant development during plant evolution [57–59]. Gene down-regulation of *FRUITFULL*-*like* homologs in other Papaveraceae like *Eschscholzia californica* and *Papaver somniferum* results in shorter fruits with premature fruit wall rupture and pericarp defects suggesting conserved roles in fruit development [60]. In addition, *Bocconia frutescens* has three *FUL*-*like* genes *BofrFL1*, *FL2* and *FL3*, from which *BofrFL2* and *BofrFL3* show expression in early carpel patterning as well as during fruit development suggesting conserved roles of *FUL*-*like* genes in fruit development across Papaveraceae [36]. On the other hand, down-regulation of *AGAMOUS*-*like* homologs in Papaveraceae results in homeotic shifts from stamen and carpel identity to petal identity accompanied by the acquisition of indeterminacy in the floral meristem [61, 62]. As gene silencing of *AG*-*like* genes block carpel development, it is unclear whether they may play roles during fruit development, and particularly if they could have conserved roles in the specification of the dehiscence zone. The fact that *BofrAG* is expressed during carpel development and maintained in early fruit development [36], together with the observation that *BofrSPT1, 2* and *3* have conserved expression patterns in comparison with their core eudicot counterparts, allow us to propose that it is likely that the upstream gene regulatory network specifying *SPT* and *ALC* expression is also maintained in basal eudicots [63].

## Conclusions

Basal eudicots are useful models to better understand the evolution of the fruit developmental network as they often carry the pre-duplication genes with respect to core eudicot model species. Although there is some redundancy between *ALC* and *SPT*, these gene clades have undergone some degree of sub-functionalization in the core eudicots. Our results also indicate that in *Bocconia frutescens*, paleo*SPT/ALC* may play a role in early floral organ specification, particularly in sepal and stamen morphogenesis that was subsequently lost in core eudicot lineages. It will be necessary to investigate the function of *paleoSPT/ALC* by knockouts or knockdowns in the Papaveraceae and do complementation assays in Arabidopsis to addressed conserved roles prior to the split of ALC and SPT clades. There is a large amount of dry fruit diversity in the Papaveraceae with diverse dehiscence mechanisms and functional studies in a range of species will help to elucidate the pre-duplication role of this gene lineage and its subsequent sub-functionalization.

## Additional files

**Additional file 1: Table S1.** List of all genes included in the phylogenetic analyses of *SPT*/*ALC* gene lineage with their respective accession number.

**Additional file 2: Figure S2.** e-values for each motif as reported in the MEME search (http://meme-suite.org/tools/meme).

**Additional file 3: Figure S1.** Alignment of the *B. frutescens* paleoSPT/ALC protein sequences. Yellow boxes highlighting the position where the probe was designed for each sequence.

**Additional file 4: Table S2.** List of the primers used for the in situ hybridization.

## Abbreviations

ALC: ALCATRAZ
bHLH: basic/helix-loop-helix
FUL: FRUITFULL
HEC: HECATE
IND: INDEHISCENT
ML: maximum likelihood
SPT: SPATULA
SHP: SHATERPROOF
WGD: whole genome duplication
