# Supplementary material for: Evolution of the SPATULA/ALCATRAZ gene lineage and expression analyses in the basal eudicot, Bocconia frutescens L. (Papaveraceae)
Source: EvoDevo. 2017 Mar 15;8:5. doi: 10.1186/s13227-017-0068-8 (PMC5353969; doi:10.1186/s13227-017-0068-8)
Supplement: Supplementary file 1 — Additional file 1: Table S1. List of all genes included in the phylogenetic analyses of SPT/ALC gene lineage with their respective accession number. [file 13227_2017_68_MOESM1_ESM.docx]

**Supplementary table 1**

Accession numbers of *SPATULA/ ALCATRAZ* *bHLH* transcription factors sequences used in this study. Most of these had been reported in Pabón-Mora et al 2014 (Supplementary table 3). New Accessions included here for the first time correspond to genbank numbers KY421362-KY421369.

|  | **Gymnosperms** |  |  |  |
| --- | --- | --- | --- | --- |
| **Gene name** | **Species** | **Family** | **Accession number** | **DataBase** |
| ***FokhoSPT*** | *Fokienia hodginsii* (Dunn) A. Henry & H.H. Thomas | Cupressaceae | UEVI-2011728 | OneKP |
| ***TetspSPT*** | *Tetraclinis sp.* | Cupressaceae | CGDN-2070388 | OneKP |
| ***CymicSPT*** | *Cycas micholitzii* Dyer | Cycadaceae | XZUY-2049748 | OneKP |
| ***MicteSPT*** | *Microcachrys tetragona (Hook.) Hook. f.* | Podocarpaceae | MHGD-2012087 | OneKP |
| ***SunamSPT*** | *Sundacarpus amarus* | Podocarpaceae | KLGF-2092082 | OneKP |
| ***TortaSPT*** | *Torreya taxifolia* | Taxaceae | EFMS-2015503 | OneKP |
|  |  |  |  |  |
|  | **Basal Angiosperms** |  |  |  |
| **Gene name** | **Species** | **Family** | **Accession number** | **DataBase** |
| ***AmtriSPT*** | *Amborella trichopoda* Baill. | Amborellaceae | URDJ-2004099 | OneKP |
| ***AfimSPT*** | *Aristolochia fimbriata* Cham. & Schltdl. | Aristolochiaceae | KY421362 | Genbank |
| ***AuscanSPT*** | *Austrobaileya scandens* C. T. White | Austrobaileyaceae | FZJL-2164770 | OneKP |
| ***AsruSPT*** | *Ascarina rubricaulis* Solms | Chloranthaceae | WZFE-2194308 | OneKP |
| ***SaglaSPT*** | *Sarcandra glabra* | Chloranthaceae | QSHQ-2009866 | OneKP |
| ***IllfloSPT*** | *Illicium floridanum* J. Ellis | Illiciaceae | VZCI-2013034 | OneKP |
|  |  |  |  |  |
|  | **Monocots** |  |  |  |
| **Gene name** | **Species** | **Family** | **Accession number** | **DataBase** |
| ***LegiSPT*** | *Lepidosperma gibsonii* | Cyperaceae | WBIB-2064717 | OneKP |
| ***HypSPT*** | *Hypoxis decumbens* L. | Hypoxidaceae | KY421366 | Genbank |
| ***OrmaSPT*** | *Orchidantha maxillarioides* (Ridl.) K. Schum. | Lowiaceae | LSKK-2019164 | OneKP |
| ***CatSPT1*** | *Cattleya trianae* Linden & Rchb. f. | Orchidaceae | KY421367 | Genbank |
| ***CatSPT2*** | *Cattleya trianae* Linden & Rchb. f. | Orchidaceae | KY421368 | Genbank |
| ***CatSPT3*** | *Cattleya trianae* Linden & Rchb. f. | Orchidaceae | KY421369 | Genbank |
| ***OrsaSPT1*** | *Oryza sativa* | Poaceae | LOC-Os06g06900 | Phytozome |
| ***OrsaSPT2*** | *Oryza sativa* | Poaceae | LOC-Os02g56140 | Phytozome |
| ***SbiSPT1*** | *Sorghum bicolor* (L.) Moench | Poaceae | Sb10g004500 | Phytozome |
| ***SbiSPT2*** | *Sorghum bicolor* (L.) Moench | Poaceae | Sb04g036450 | Phytozome |
| ***ZemaSPT1*** | *Zea mays* L. | Poaceae | GRMZM2G017349 | Phytozome |
| ***ZemaSPT2*** | *Zea mays* L. | Poaceae | GRMZM2G030744 | Phytozome |
|  |  |  |  |  |
|  | **Basal Eudicots** |  |  |  |
| **Gene name** | **Species** | **Family** | **Accession number** | **DataBase** |
| ***AktriALC*** | *Akebia trilobata* | Lardizabalaceae | CCID-2010152 | OneKP |
| ***ArmeSPT*** | *Argemone mexicana* L. | Papaveraceae | IRAF-2111869 | OneKP |
| ***BofrSPT1*** | *Bocconia frutescens* L. | Papaveraceae | KY421363 | Genbank |
| ***BofrSPT2*** | *Bocconia frutescens* L. | Papaveraceae | KY421364 | Genbank |
| ***BofrSPT3*** | *Bocconia frutescens* L. | Papaveraceae | KY421365 | Genbank |
| ***EscaSPT*** | *Eschscholzia californica* | Papaveraceae | EVOD-2110824 | OneKP |
| ***PaseSPT*** | *Papaver setigerum* | Papaveraceae | MLPX-2019231 | OneKP |
| ***PsomSPT*** | *Papaver somniferum* L. | Papaveraceae | RQNK-2019222 | OneKP |
| ***AquSPT*** | *Aquilegia coerulea* E. James | Ranunculaceae | Aquca-007-007-02 | Phytozome |
|  |  |  |  |  |
|  | **Core Eudicots** |  |  |  |
| **Gene name** | **Species** | **Family** | **Accession number** | **DataBase** |
| ***AssyALC*** | *Asclepias syriaca* L. | Asclepiadaceae | YADI-2014020 | OneKP |
| ***AssySPT*** | *Asclepias syriaca* L. | Asclepiadaceae | YADI*-*2010022 | OneKP |
| ***AlyrALC*** | *Arabidopsis lyrata* (L.) O'Kane & Al-Shehbaz | Brassicaceae | 496889 | Phytozome |
| ***AlyrSPT*** | *Arabidopsis lyrata* (L.) O'Kane & Al-Shehbaz | Brassicaceae | 353119 | Phytozome |
| ***ALC*** | *Arabidopsis thaliana* (L.) Heynh. | Brassicaceae | AT5G67110 | Phytozome |
| ***SPT*** | *Arabidopsis thaliana* (L.) Heynh. | Brassicaceae | AT4G36930 | Phytozome |
| ***BraALC*** | *Brassica rapa* | Brassicaceae | Bra012133 | Phytozome |
| ***BraSPT1*** | *Brassica rapa* | Brassicaceae | Bra011740 | Phytozome |
| ***BraSPT2*** | *Brassica rapa* | Brassicaceae | Bra010591 | Phytozome |
| ***CaruALC*** | *Capsella rubella* (E.B. Almq.) E.B. Almq. | Brassicaceae | Carubv10028570m | OneKP |
| ***CaruSPT*** | *Capsella rubella* (E.B. Almq.) E.B. Almq. | Brassicaceae | Carubv10005083m | OneKP |
| ***RicoALC*** | *Ricinus communis* L. | Euphorbiaceae | 30170.t000264 | Phytozome |
| ***RicoSPT*** | *Ricinus communis* L. | Euphorbiaceae | 30115.t.000061 | Phytozome |
| ***MetrALC*** | *Medicago truncatula* | Fabaceae | Medtr1g019240 | Phytozome |
| ***MetrSPT**** | *Medicago truncatula* | Fabaceae | Medtr5g017040 | Phytozome |
| ***MiguALC1**** | *Mimulus guttatus* | Phrymaceae | Mgv.1a010846m.g | Phytozome |
| ***PotriALC~*** | *Populus trichocarpa* | Salicaceae | Potri014G025800 | Phytozome |
| ***PotriSPT~*** | *Populus trichocarpa* | Salicaceae | Potri005G139700 | Phytozome |
| ***NsylALC*** | *Nicotiana sylvestris* Speg. & S. Comes | Solanaceae | MKZR-2027110 | OneKP |
| ***NsylSPT*** | *Nicotiana sylvestris* Speg. & S. Comes | Solanaceae | MKZR-2026090 | OneKP |
| ***SlyALC**** | *Solanum lycopersicum* | Solanaceae | Solyc03g0444460 | Phytozome |
| ***SlySPT**** | *Solanum lycopersicum* | Solanaceae | Solyc02g093280.2 | Phytozome |
| ***ThecALC**** | *Theobroma cacao* | Sterculiaceae | Thecc1EG033802t.1 | Phytozome |
| ***ThecSPT1**** | *Theobroma cacao* | Sterculiaceae | Thecc1EG000649t.1 | Phytozome |
| ***ViviALC~*** | *Vitis vinifera* | Vitaceae | GSVIVT01009467001 | Phytozome |
| ***ViviSPT~*** | *Vitis vinifera* | Vitaceae | GSVIVG0102211001 | Phytozome |
