# Supplementary figures and images for: Evolution of the SPATULA/ALCATRAZ gene lineage and expression analyses in the basal eudicot, Bocconia frutescens L. (Papaveraceae)
Source: EvoDevo. 2017 Mar 15;8:5. doi: 10.1186/s13227-017-0068-8 (PMC5353969; doi:10.1186/s13227-017-0068-8)

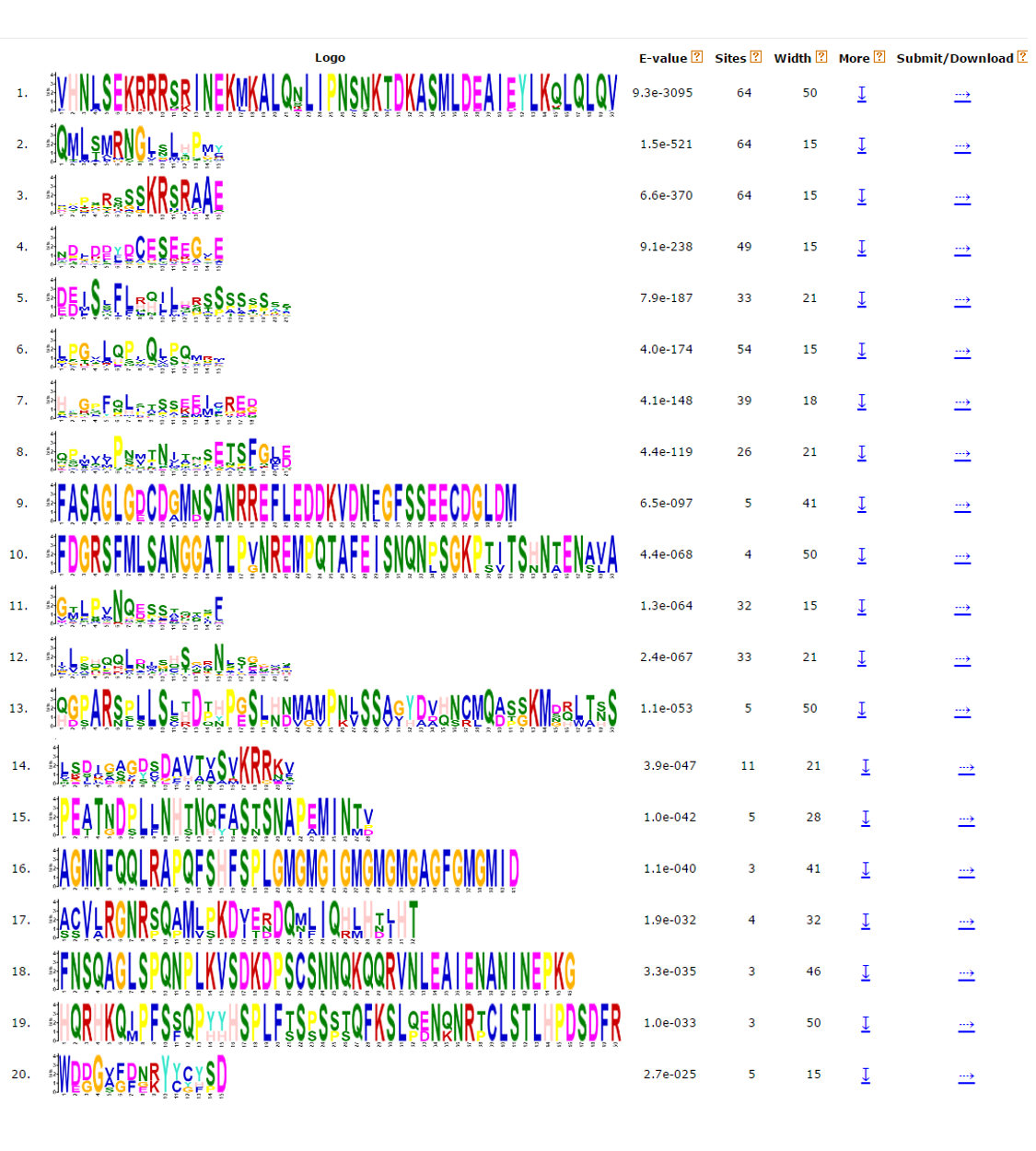

Supplement: Supplementary file 2 — Additional file 2: Figure S2. e-values for each motif as reported in the MEME search (http://meme-suite.org/tools/meme). [file 13227_2017_68_MOESM2_ESM.tif]

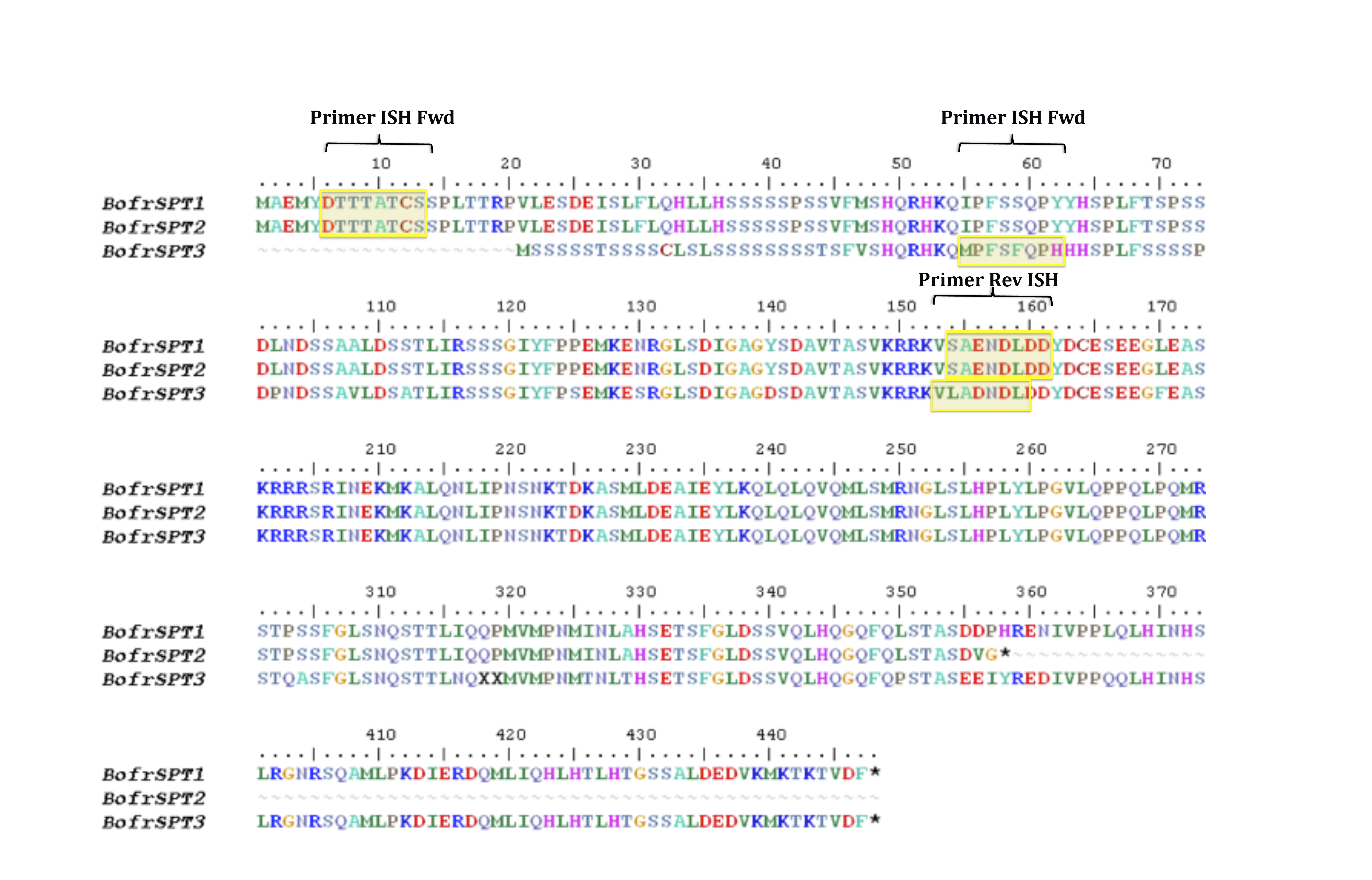

Supplement: Supplementary file 3 — Additional file 3: Figure S1. Alignment of the B. frutescens paleoSPT/ALC protein sequences. Yellow boxes highlighting the position where the probe was designed for each sequence. [file 13227_2017_68_MOESM3_ESM.tif]
