# Supplementary material for: Evolution of the SPATULA/ALCATRAZ gene lineage and expression analyses in the basal eudicot, Bocconia frutescens L. (Papaveraceae)
Source: EvoDevo. 2017 Mar 15;8:5. doi: 10.1186/s13227-017-0068-8 (PMC5353969; doi:10.1186/s13227-017-0068-8)
Supplement: Supplementary file 4 — Additional file 4: Table S2. List of the primers used for the in situ hybridization. [file 13227_2017_68_MOESM4_ESM.docx]

**Supplementary table 2**

Primers used for ISH probe design. as indicates antisense probe.

| ***BofrSPT1/2* Fwd ISH as** | GACACCACCACCGCCACATGTT |
| --- | --- |
| ***BofrSPT1/2* Rev ISH as** | CTTAATACGACTCACTATAGGGATCATCAAGATCATTTTCCGCCG |
| ***BofrSPT3* Fwd ISH as** | GCCCTTTTCATTTCAACCCC |
| ***BofrSPT3* Rev ISH as** | CTTAATACGACTCACTATAGGGGAGATCGTTATCAGCCAGGAC |
| ***BofrSPT1/2* Fwd ISH sense** | CTTAATACGACTCACTATAGGGGACACCACCACCGCCACATGTT |
| ***BofrSPT1/2* Rev ISH sense** | ATCATCAAGATCATTTTCCGCCG |
| ***BofrSPT3* Fwd ISH sense** | CTTAATACGACTCACTATAGGGGCCCTTTTCATTTCAACCCC |
| ***BofrSPT3* Rev ISH sense** | GAGATCGTTATCAGCCAGGAC |
